# Supplementary material for: healthcareCOVID: a national cross-sectional observational study identifying risk factors for developing suspected or confirmed COVID-19 in UK healthcare workers
Source: PeerJ. 2021 Feb 4;9:e10891. doi: 10.7717/peerj.10891 (PMC7868068; doi:10.7717/peerj.10891)
Supplement: Supplemental Information 2 — Contains: (1) overview of healthcareCOVID survey, (2) STROBE checklist, (3) Disclaimer for healthcareCOVID survey, and (4) eFigures 1 - 4 and eTables 1 - 10. [file peerj-09-10891-s002.docx]

*Title*: **healthcareCOVID: A national cross-sectional observational study identifying risk factors for developing suspected or confirmed COVID-19 in UK healthcare workers – SUPPLEMENT MATERIAL**

**Supplement material**

- **APPENDIX 1** - healthcareCOVID survey overview
  - [Note: Ethnicity layout as per UK government^1^]
- **APPENDIX 2** - STrengthening the Reporting of OBservational studies in Epidemiology (STROBE) checklist^2^ for healthcareCOVID
- **APPENDIX 3** - Disclaimer for healthcareCOVID survey
- **eFigure 1** - Bar graph reporting the areas of clinical contact with suspected or confirmed COVID-19 patients by participants reporting regular clinical contact (n = 3902)
- **eFigure 2** - Bar graph of exposure to aerosol-generating procedures (AGPs) performed in suspected or confirmed COVID-19 patients amongst participants who had regular exposure (n = 2296)
- **eTable 1** - Doctors by Specialty
- **eTable 2** - Doctors by Grade
- **eTable 3** - Non-doctors by Specialty
- **eTable 4** - Non-doctors by Grade
- **eFigure 3** - Bar graph of symptoms reported by participants during self-isolation due to symptoms or testing positive for SARS-CoV-2 (n = 1,776)
- **eFigure 4** - Bar graph of reasons for inadequate PPE use during clinical contact with suspected/confirmed COVID-19 patients of those who had been in situations where they did not use adequate PPE (n = 1306)
- **eTable 5** - Numbers (%) for all outcomes and composite outcome
- **eTable 6** - Multivariable logistic regression model featuring constituent BAME groups
- **eTable 7** - Multivariable logistic regression model with comorbidities removed to assess for collider bias with respect to smoking status
- **eTable 8** - Multivariable logistic regression model including clinical areas in which healthcare workers had regular clinical contact with suspected or confirmed COVID-19 patients
- **eTable 9** - Multivariable logistic regression model including AGPs to which healthcare workers were regularly exposed to when performed in suspected or confirmed COVID-19 patients
- **eTable10** - Multivariable logistic regression model with the outcome being participants stating that they had laboratory-confirmed SARS-CoV-2 infection (via reverse transcription polymerase chain reaction or antibody testing)

**References**:

1. GOV.UK. Ethnic groups. Accessed July 9, 2020. https://design-system.service.gov.uk/patterns/ethnic-group/

2. STROBE Group. STROBE Statement: Available checklists. Accessed July 5, 2020. https://www.strobe-statement.org/index.php?id=available-checklists

**APPENDIX 1** – healthcareCOVID survey overview

The following is an overview of the questions and data collected from the online survey hosted at <https://intubatecovid.knack.com/healthcarecovid>:

*Entry Validation*

- E-mail Address
  - Survey validation check to reduce number of duplicate entries from the same participant
  - *Not* linked to responses during data analysis

*Demographics (Section 1 of 5)*

- Age
  - [Free-text]
- Sex assigned at birth
  - Male
  - Female
- Does your gender match the sex you were assigned at birth?
  - Yes
  - No
    - Conditional 🡪 If "No", please specify:
      - [Free-text]
- Ethnicity
  - As per layout from UK government^1^
- Household environment
  - How many people live in your household, including yourself?
    - I live alone
    - I live with 1 or more persons
      - Conditional 🡪 Do you have children living at home with you?
        - Yes
        - No
- Health Status
  - Do you have any of the following health problems, even if treated or under control? (*optional*)
    - 11 options with *tick all that apply* approach
  - Tobacco smoking status (optional):
    - Current smoker or Ex-smoker (within 1 year)
    - Ex-smoker (more than 1 year)
    - Never smoked

*Work Details (Section 2 of 5)*

- Country
  - England
  - Northern Ireland
  - Scotland
  - Wales
- County/Region
  - Drop-down list based on Country input
- Main healthcare facility
  - Hospital
  - Community healthcare facility
  - Social care facility
  - Other
- Role
  - Drop-down list with 32 options
- Grade
  - Drop-down list with 16 options
- Primary specialty
  - Drop-down list based on Role input

*COVID-19 status (Section 3 of 5)*

Since 1^st^ February 2020…

- Have you ever self-isolated because of COVID-19 symptoms and/or had a positive test?
  - Yes
    - Conditional 🡪 How long did you self-isolate?
      - 1 to 7 days
      - 8 to 14 days
      - More than 14 days
        - Conditional 🡪 How long? (in days)

[Free-text]

- - - Conditional 🡪 Have you had to self-isolate more than once because of COVID-19 symptoms?
      - Yes
      - No
    - Conditional 🡪 What symptoms did you have during self-isolation?
      - 13 options with *tick all that apply* approach
- Have you ever been hospitalised due to having suspected or confirmed COVID-19?
  - Yes
  - No
- Have you ever tested POSITIVE for COVID-19?
  - Yes - Oral/Nasal swab
  - Yes - Blood test
  - No - I have NOT tested positive (e.g. tests all negative, awaiting results)
  - No - I have NEVER BEEN tested

*Exposure characteristics (Section 4 of 5)*

Since 1^st^ Feb 2020…

- Do you regularly use public transport to travel to work?
  - Yes
  - No
- During your usual work shift, have you had regular clinical contact with suspected or confirmed COVID-19 patients?
  - Yes
    - Conditional 🡪 If "Yes" in what areas/environments?
      - 15 options with *tick all that apply* approach
  - No
- During your usual work shift, have you been regularly exposed to aerosol generating procedures (AGPs) performed in suspected or confirmed COVID-19 patients?
  - Yes
    - Conditional 🡪 If "Yes", what AGPs?
      - 13 options with *tick all that apply* approach

*Personal protective equipment (PPE) use (Section 5 of 5)*

Since 1^st^ Feb 2020…

- Have you had sufficient training in PPE usage?
  - Yes
  - No
- Have you had any episodes where you LACKED ACCESS to PPE items when having clinical contact with suspected or confirmed COVID-19 patients?
  - Yes
    - Conditional 🡪 If "Yes", what PPE items did you LACK ACCESS to?
      - 9 options with *tick all that apply* approach
  - No
- Have you had any clinical contact with suspected or confirmed cases of COVID-19 where you DID NOT use adequate PPE?
  - Yes
    - Conditional 🡪 If "Yes", please select:
      - Always
      - Often
      - Sometimes
      - Rarely
    - Conditional 🡪 What was the reason(s) for NOT using adequate PPE during these encounter(s)?
      - 7 options with *tick all that apply* approach
- Have you ever had to reuse disposable PPE items?
  - Yes
  - No
- Have you ever had to improvise or make your own PPE items?
  - Yes
  - No

*Other comments*

- Is there anything else you would like to let us know?
  - [Free-text]

Checkbox option for participant to be contacted in future regarding further information about the survey (optional)

**APPENDIX 2** – STrengthening the Reporting of OBservational studies in Epidemiology (STROBE) checklist^1^ for healthcareCOVID

**STROBE Statement—Checklist of items that should be included in reports of *cross-sectional studies***

|  | **Item No** | **Recommendation** | **Recorded on Page No.** |
| --- | --- | --- | --- |
| **Title and abstract** | 1 | (*a*) Indicate the study’s design with a commonly used term in the title or the abstract | pg. 1 |
|  |  | (*b*) Provide in the abstract an informative and balanced summary of what was done and what was found | pg. 2 – 3 |
| **Introduction** | | |  |
| Background/rationale | 2 | Explain the scientific background and rationale for the investigation being reported | pg. 3 – 5 |
| Objectives | 3 | State specific objectives, including any prespecified hypotheses | pg. 5 |
| **Methods** | | |  |
| Study design | 4 | Present key elements of study design early in the paper | pg. 5 – 10 |
| Setting | 5 | Describe the setting, locations, and relevant dates, including periods of recruitment, exposure, follow-up, and data collection | pg. 5 – 7 |
| Participants | 6 | (*a*) Give the eligibility criteria, and the sources and methods of selection of participants | pg. 5 – 6 |
| Variables | 7 | Clearly define all outcomes, exposures, predictors, potential confounders, and effect modifiers. Give diagnostic criteria, if applicable | pg. 8 – 9 |
| Data sources/ measurement | 8* | For each variable of interest, give sources of data and details of methods of assessment (measurement). Describe comparability of assessment methods if there is more than one group | ***Appendix 1***  (Supplement material) |
| Bias | 9 | Describe any efforts to address potential sources of bias | pg. 18 – 19 |
| Study size | 10 | Explain how the study size was arrived at | N/A |
| Quantitative variables | 11 | Explain how quantitative variables were handled in the analyses. If applicable, describe which groupings were chosen and why | pg. 8 – 9 |
| Statistical methods | 12 | (*a*) Describe all statistical methods, including those used to control for confounding | pg. 8 – 9 |
|  |  | (*b*) Describe any methods used to examine subgroups and interactions | pg. 10 |
|  |  | (*c*) Explain how missing data were addressed | pg. 9 |
|  |  | (*d*) If applicable, describe analytical methods taking account of sampling strategy | pg. 8 – 9 |
|  |  | (*e*) Describe any sensitivity analyses | N/A |
| **Results** | | |  |
| Participants | 13* | (a) Report numbers of individuals at each stage of study—eg numbers potentially eligible, examined for eligibility, confirmed eligible, included in the study, completing follow-up, and analysed | ***Figure 1*** |
|  |  | (b) Give reasons for non-participation at each stage | ***Figure 1*** |
|  |  | (c) Consider use of a flow diagram | ***Figure 1*** |
| Descriptive data | 14* | (a) Give characteristics of study participants (eg demographic, clinical, social) and information on exposures and potential confounders | ***Table 1*** |
|  |  | (b) Indicate number of participants with missing data for each variable of interest | N/A |
| Outcome data | 15* | Report numbers of outcome events or summary measures | ***eTable 5***  (Supplement material) |
| Main results | 16 | (*a*) Give unadjusted estimates and, if applicable, confounder-adjusted estimates and their precision (eg, 95% confidence interval). Make clear which confounders were adjusted for and why they were included | ***Table 2*** |
|  |  | (*b*) Report category boundaries when continuous variables were categorized | N/A |
|  |  | (*c*) If relevant, consider translating estimates of relative risk into absolute risk for a meaningful time period | N/A |
| Other analyses | 17 | Report other analyses done—eg analyses of subgroups and interactions, and sensitivity analyses | pg. 13 – 14, ***eFigures 1 – 4*** *and* ***eTables 6 – 10*** (Supplement material) |
| **Discussion** | | |  |
| Key results | 18 | Summarise key results with reference to study objectives | pg. 14 – 15 |
| Limitations | 19 | Discuss limitations of the study, taking into account sources of potential bias or imprecision. Discuss both direction and magnitude of any potential bias | pg. 18 – 19 |
| Interpretation | 20 | Give a cautious overall interpretation of results considering objectives, limitations, multiplicity of analyses, results from similar studies, and other relevant evidence | pg. 14 – 20 |
| Generalisability | 21 | Discuss the generalisability (external validity) of the study results | pg. 19 – 20 |
| **Other information** | | |  |
| Funding | 22 | Give the source of funding and the role of the funders for the present study and, if applicable, for the original study on which the present article is based | No specific grant from any funding agency in the public, commercial, or not-for-profit sectors. |

*Give information separately for exposed and unexposed groups.

**Note:** An Explanation and Elaboration article discusses each checklist item and gives methodological background and published examples of transparent reporting. The STROBE checklist is best used in conjunction with this article (freely available on the Web sites of PLoS Medicine at http://www.plosmedicine.org/, Annals of Internal Medicine at http://www.annals.org/, and Epidemiology at http://www.epidem.com/). Information on the STROBE Initiative is available at www.strobe-statement.org.

**APPENDIX 3** – Disclaimer for healthcareCOVID survey

**Disclaimer**

The healthcareCOVID project has been registered as a service evaluation (ID 10834) at Guy's and St Thomas' NHS Foundation Trust (London, UK).

By submitting my responses, I confirm that:

1. I understand the purpose of this survey.
2. I have freely chosen to participate.
3. I understand that there will be no reimbursement for my participation.
4. I understand that the principal purpose of this project is to prevent disease transmission and to improve public health services. I understand that communication of findings from this project may involve presentation or publication of collected data and that all data will be de-identified and reported in aggregate.
5. I understand that my data will be stored in an online encrypted database accessed only by the healthcareCOVID team, and in accordance with GDPR principles, and that no personal information will be shared with any third party.
6. I will receive information as requested, only if I have confirmed as such on the survey.

**
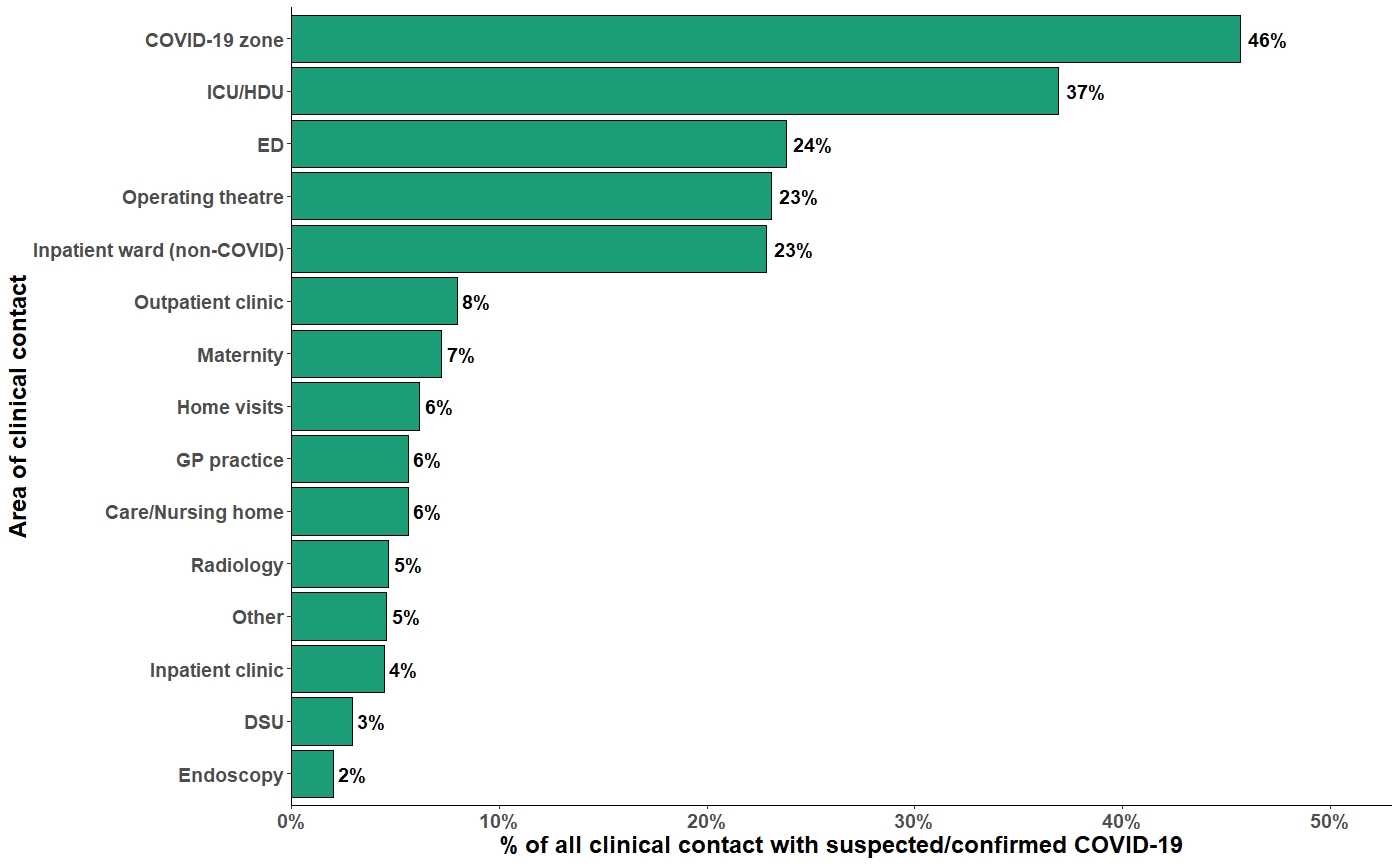
**

**eFigure 1 Bar graph reporting the areas of clinical contact with suspected or confirmed covid-19 patients by participants reporting regular clinical contact (n = 3902).**

**
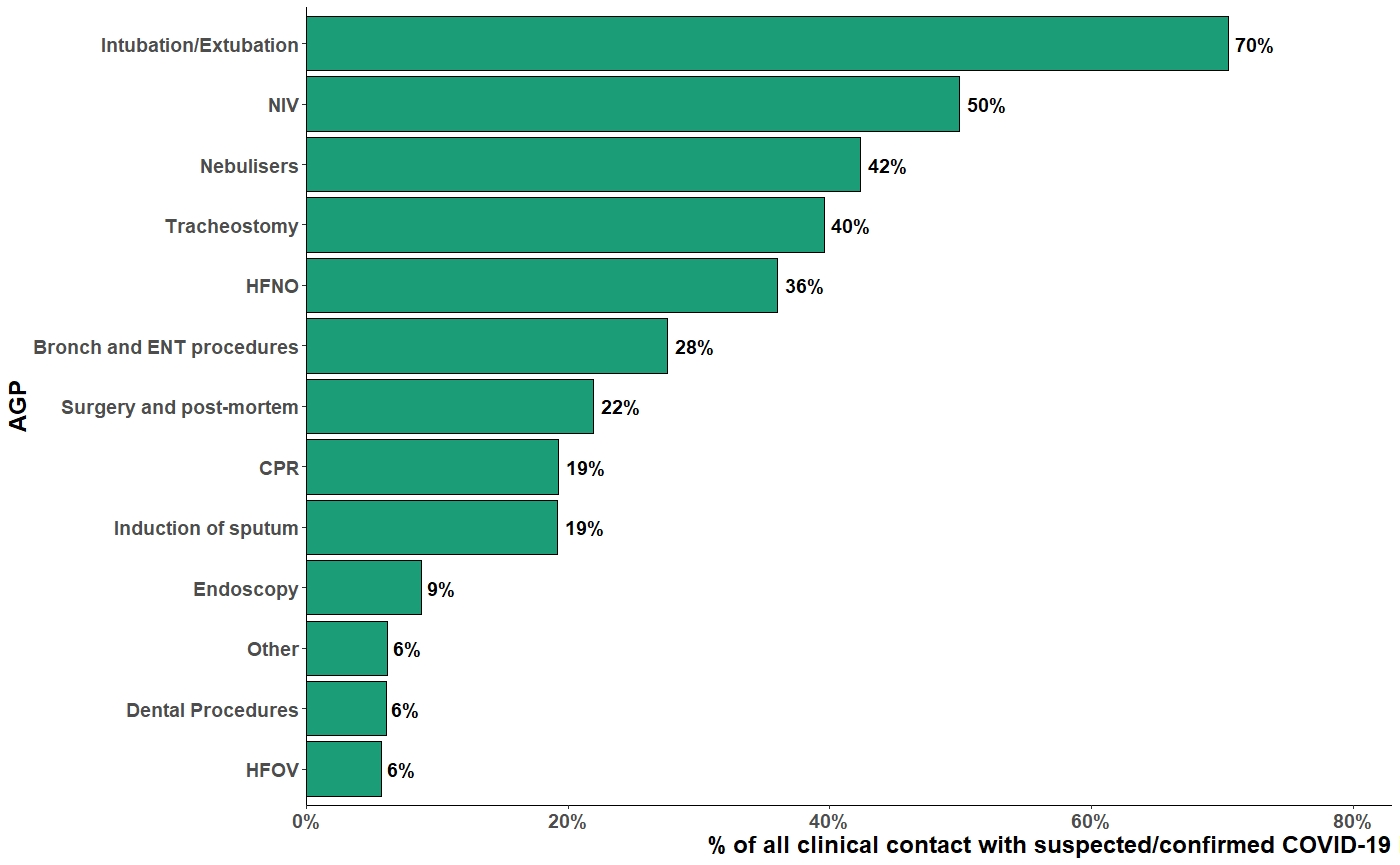
**

**eFigure 2 Bar graph of exposure to aerosol-generating procedures (AGPs) performed in suspected or confirmed covid-19 patients amongst participants who had regular exposure (n = 2296).**

*Abbreviations*: NIV = Non-invasive ventilation; HFNO = High-flow nasal oxygen; Bronch = Bronchoscopy; ENT = Ear, Nose and Throat; CPR = Cardiopulmonary resuscitation; HFOV = High Frequency Oscillatory Ventilation.

**eTable 1. Doctors by specialty**

| **Specialty** | **n** | **%** |
| --- | --- | --- |
| Acute internal medicine | 52 | 2.9 |
| Allergy | 1 | 0.1 |
| Anaesthetics | 406 | 23.0 |
| Cardio-thoracic surgery | 2 | 0.1 |
| Cardiology | 23 | 1.3 |
| Chemical pathology | 2 | 0.1 |
| Child and adolescent psychiatry | 5 | 0.3 |
| Clinical genetics | 1 | 0.1 |
| Clinical oncology | 11 | 0.6 |
| Clinical pharmacology and therapeutics | 1 | 0.1 |
| Clinical radiology | 20 | 1.1 |
| Dermatology | 12 | 0.7 |
| Emergency medicine | 96 | 5.4 |
| Endocrinology and diabetes mellitus | 14 | 0.8 |
| Forensic histopathology | 1 | 0.1 |
| Forensic psychiatry | 1 | 0.1 |
| Gastroenterology | 16 | 0.9 |
| General (internal) medicine | 40 | 2.3 |
| General practice | 403 | 22.8 |
| General psychiatry | 22 | 1.2 |
| General surgery | 38 | 2.1 |
| Genitourinary medicine | 7 | 0.4 |
| Geriatric medicine | 51 | 2.9 |
| Haematology | 20 | 1.1 |
| Histopathology | 12 | 0.7 |
| Immunology | 2 | 0.1 |
| Infectious diseases | 9 | 0.5 |
| Intensive care medicine | 129 | 7.3 |
| Medical microbiology | 4 | 0.2 |
| Medical oncology | 6 | 0.3 |
| Neurology | 9 | 0.5 |
| Neurosurgery | 3 | 0.2 |
| Obstetrics and gynaecology | 62 | 3.5 |
| Occupational medicine | 1 | 0.1 |
| Old age psychiatry | 11 | 0.6 |
| Ophthalmology | 25 | 1.4 |
| Oral and maxillofacial surgery | 7 | 0.4 |
| OTHER | 11 | 0.6 |
| Otolaryngology | 25 | 1.4 |
| Paediatric and perinatal pathology | 1 | 0.1 |
| Paediatric cardiology | 1 | 0.1 |
| Paediatric surgery | 2 | 0.1 |
| Paediatrics | 72 | 4.1 |
| Palliative medicine | 11 | 0.6 |
| Plastic surgery | 4 | 0.2 |
| Psychiatry of learning disability | 2 | 0.1 |
| Public health medicine | 2 | 0.1 |
| Rehabilitation medicine | 1 | 0.1 |
| Renal medicine | 8 | 0.5 |
| Respiratory medicine | 32 | 1.8 |
| Rheumatology | 12 | 0.7 |
| Trauma and orthopaedic surgery | 34 | 1.9 |
| Urology | 15 | 0.8 |
| Vascular surgery | 11 | 0.6 |

**eTable 2. Doctors by grade**

| **Grade** | **n** | **%** |
| --- | --- | --- |
| Clinical Fellow | 38 | 2.1 |
| Consultant | 776 | 43.8 |
| CT/ACCS/ST1-2 or Trust Grade equivalent | 130 | 7.3 |
| Foundation Year | 67 | 3.8 |
| GP (Sessional/Salaried/Partner) | 357 | 20.2 |
| OTHER | 9 | 0.5 |
| SpR (ST3+) or Trust Grade equivalent | 323 | 18.2 |
| Staff/Associate Specialist | 69 | 3.9 |
| Student | 1 | 0.1 |

**eTable 3. Specialties associated with working roles for healthcare workers other than doctors**

| **Specialty** | **n** | **%** |
| --- | --- | --- |
| Dentistry | 161 | 3.7 |
| Medical | 764 | 17.4 |
| Mental Health | 197 | 4.5 |
| OTHER | 1306 | 29.8 |
| Paediatrics | 183 | 4.2 |
| Pharmacy | 81 | 1.8 |
| Prehospital & Emergency Services | 163 | 3.7 |
| Primary Care & Public Health | 448 | 10.2 |
| Surgical | 930 | 21.2 |
| Women's Health | 149 | 3.4 |

**eTable 4. Grades for healthcare workers other than doctors**

| **Grade** | **n** | **%** |
| --- | --- | --- |
| Band 2 | 115 | 2.6 |
| Band 3 | 124 | 2.8 |
| Band 4 | 132 | 3.0 |
| Band 5 | 795 | 18.1 |
| Band 6 | 1162 | 26.5 |
| Band 7 | 881 | 20.1 |
| Band 8+ | 588 | 13.4 |
| Consultant | 33 | 0.8 |
| CT/ACCS/ST1-2 or Trust Grade equivalent | 4 | 0.1 |
| Foundation Year | 3 | 0.1 |
| GP (Sessional/Salaried/Partner) | 44 | 1.0 |
| OTHER | 389 | 8.9 |
| SpR (ST3+) or Trust Grade equivalent | 3 | 0.1 |
| Staff/Associate Specialist | 63 | 1.4 |
| Student | 46 | 1.0 |

**
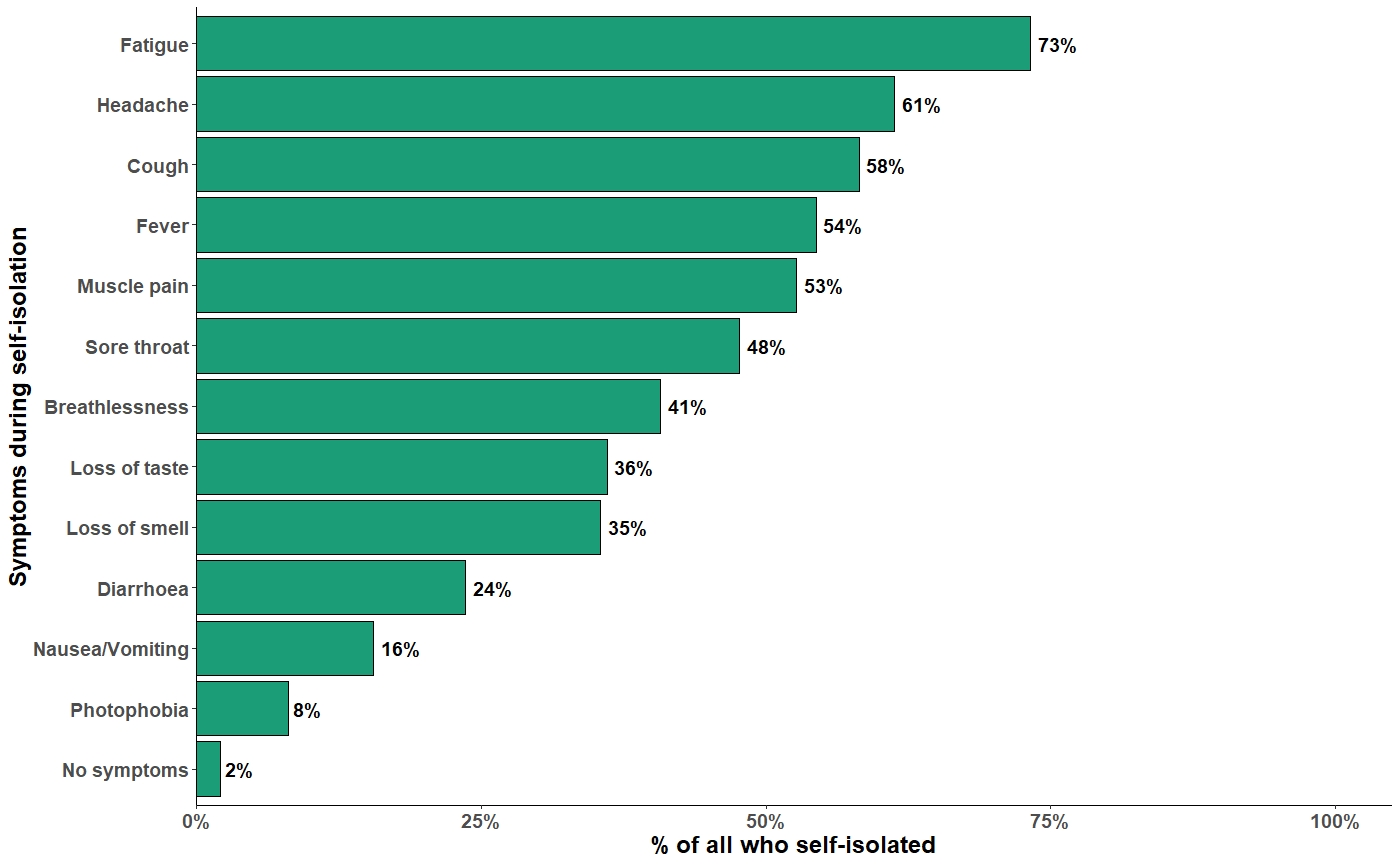
**

**eFigure 3 Bar graph of symptoms reported by participants during self-isolation due to symptoms or testing positive for SARS-CoV-2 (n = 1,776).**


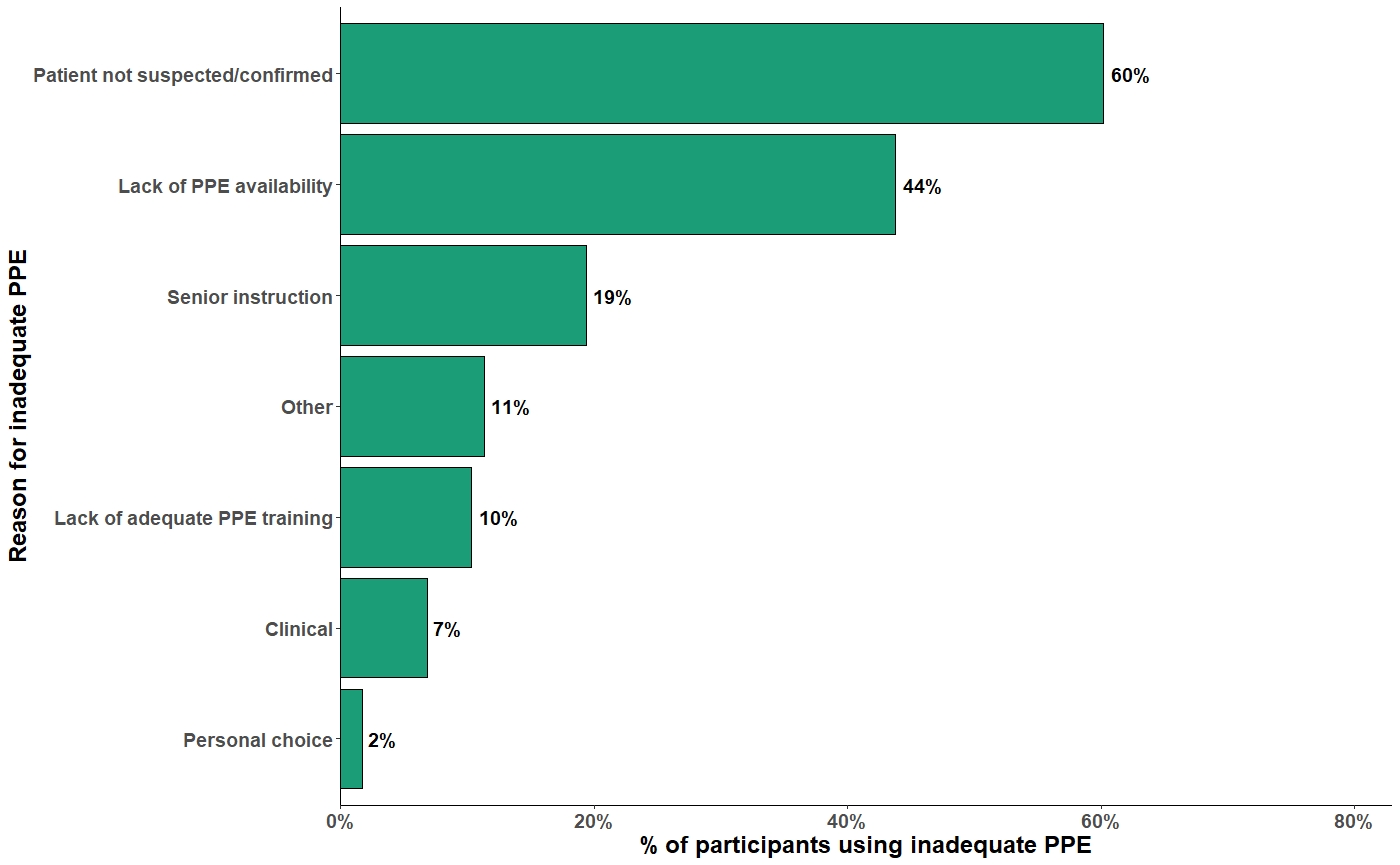


**eFigure 4 Bar graph of reasons for inadequate PPE use during clinical contact with suspected/confirmed COVID-19 patients of those who had been in situations where they did not use adequate PPE (n = 1306).**

|  | **Self-isolation** | | **Hospitalised** | | **COVID-19 positive** | | **COVID-19 Composite** | |
| --- | --- | --- | --- | --- | --- | --- | --- | --- |
|  | n | % | n | % | n | % | n | % |
|  | 1776 | 28.9 | 49 | 0.80 | 459 | 7.46 | 1806 | 29.4 |
| **Age** | | | | | | | | |
| *years, mean (SD)* | 41.9 (10.2) | | 42.6 (11.9) | | 42.5 (10.2) | | 41.9 (10.2) | |
| **Sex** | | | | | | | | |
| *Male* | 379 | 6.2 | 17 | 0.3 | 126 | 2 | 390 | 6.3 |
| *Female* | 1397 | 22.7 | 32 | 0.5 | 333 | 5.4 | 1416 | 23 |
| **Ethnicity** | | | | | | | | |
| *White* | 1313 | 21.3 | 29 | 0.5 | 331 | 5.4 | 1330 | 21.6 |
| *BAME* | 455 | 7.4 | 20 | 0.3 | 127 | 2.1 | 468 | 7.6 |
| *Prefer not to say* | 8 | 0.1 | 0 | 0 | 1 | 0 | 8 | 0.1 |
| **Household** | | | | | | | | |
| *Lives alone* | 200 | 3.3 | 6 | 0.1 | 49 | 0.8 | 205 | 3.3 |
| *Lives with 1 or more persons; no children* | 654 | 10.6 | 25 | 0.4 | 168 | 2.7 | 664 | 10.8 |
| *Lives with 1 or more persons; has children* | 922 | 15 | 18 | 0.3 | 242 | 3.9 | 937 | 15.2 |
| **Comorbidities** | | | | | | | | |
| *Hypertension* | 157 | 2.6 | 6 | 0.1 | 35 | 0.6 | 158 | 2.6 |
| *Diabetes* | 54 | 0.9 | 6 | 0.1 | 17 | 0.3 | 57 | 0.9 |
| *Cancer* | 28 | 0.5 | 0 | 0 | 5 | 0.1 | 28 | 0.5 |
| *Heart disease* | 23 | 0.4 | 0 | 0 | 4 | 0.1 | 23 | 0.4 |
| *Immunosuppression* | 27 | 0.4 | 2 | 0 | 2 | 0 | 27 | 0.4 |
| *Respiratory disease* | 192 | 3.1 | 15 | 0.2 | 49 | 0.8 | 198 | 3.2 |
| *Renal disease* | 10 | 0.2 | 2 | 0 | 3 | 0 | 10 | 0.2 |
| *Liver disease* | 10 | 0.2 | 0 | 0 | 2 | 0 | 10 | 0.2 |
| *Neurological disease* | 18 | 0.3 | 0 | 0 | 3 | 0 | 18 | 0.3 |
| *Obesity* | 234 | 3.8 | 8 | 0.1 | 47 | 0.8 | 236 | 3.8 |
| *Prefer not to say* | 0 | 0.4 | 0 | 0 | 0 | 0.1 | 0 | 0.4 |
| **Smoking status** | | | | | | | | |
| *Never smoked* | 1258 | 20.4 | 38 | 0.6 | 327 | 5.3 | 1279 | 20.8 |
| *Current or Ex-smoker within 1 year* | 139 | 2.3 | 3 | 0 | 27 | 0.4 | 142 | 2.3 |
| *Ex-smoker > 1 year* | 357 | 5.8 | 7 | 0.1 | 96 | 1.6 | 361 | 5.9 |
| *Prefer not to say* | 22 | 0.4 | 1 | 0 | 9 | 0.1 | 24 | 0.4 |
| **Country** | | | | | | | | |
| *England* | 1615 | 26.3 | 49 | 0.8 | 419 | 6.8 | 1645 | 26.7 |
| *Northern Ireland* | 15 | 0.2 | 0 | 0 | 6 | 0.1 | 15 | 0.2 |
| *Scotland* | 89 | 1.4 | 0 | 0 | 18 | 0.3 | 89 | 1.4 |
| *Wales* | 57 | 0.9 | 0 | 0 | 16 | 0.3 | 57 | 0.9 |
| **Main Healthcare Facility** | | | | | | | | |
| *Hospital* | 1331 | 21.6 | 38 | 0.6 | 375 | 6.1 | 1356 | 22 |
| *Community healthcare facility* | 316 | 5.1 | 7 | 0.1 | 56 | 0.9 | 319 | 5.2 |
| *Social care facility* | 48 | 0.8 | 1 | 0 | 16 | 0.3 | 49 | 0.8 |
| *Other* | 81 | 1.3 | 3 | 0 | 12 | 0.2 | 82 | 1.3 |
| **Role Group** | | | | | | | | |
| *Nurses, midwives and associated staff* | 752 | 12.2 | 16 | 0.3 | 194 | 3.2 | 759 | 12.3 |
| *Allied health professionals* | 274 | 4.5 | 9 | 0.1 | 54 | 0.9 | 280 | 4.6 |
| *Dentists and dental staff* | 28 | 0.5 | 0 | 0 | 2 | 0 | 29 | 0.5 |
| *Doctors* | 578 | 9.4 | 20 | 0.3 | 172 | 2.8 | 592 | 9.6 |
| *Other* | 144 | 2.3 | 4 | 0.1 | 37 | 0.6 | 146 | 2.4 |
| **Used public transport to travel to work** | | | | | | | | |
| *No* | 1343 | 21.8 | 38 | 0.6 | 362 | 5.9 | 1363 | 22.2 |
| *Yes* | 433 | 7 | 11 | 0.2 | 97 | 1.6 | 443 | 7.2 |
| **Regular clinical contact with suspected or confirmed COVID-19 patients** | | | | | | | | |
| *No* | 533 | 8.7 | 10 | 0.2 | 88 | 1.4 | 540 | 8.8 |
| *Yes* | 1243 | 20.2 | 39 | 0.6 | 371 | 6 | 1266 | 20.6 |
| **Regular exposure to AGP(s) performed in suspected or confirmed COVID-19 patients** | | | | | | | | |
| *No* | 1129 | 18.4 | 29 | 0.5 | 289 | 4.7 | 1142 | 18.6 |
| *Yes* | 647 | 10.5 | 20 | 0.3 | 170 | 2.8 | 664 | 10.8 |
| **Sufficient training in PPE use** | | | | | | | | |
| *No* | 591 | 9.6 | 16 | 0.3 | 130 | 2.1 | 598 | 9.7 |
| *Yes* | 1185 | 19.3 | 33 | 0.5 | 329 | 5.3 | 1208 | 19.6 |
| **Lacked access to PPE items for clinical contact with suspected or confirmed COVID-19 patients** | | | | | | | | |
| *No* | 1246 | 20.3 | 32 | 0.5 | 297 | 4.8 | 1271 | 20.7 |
| *Yes* | 530 | 8.6 | 17 | 0.3 | 162 | 2.6 | 535 | 8.7 |
| **Clinical contact without adequate PPE** | | | | | | | | |
| *Never* | 1258 | 20.4 | 31 | 0.5 | 299 | 4.9 | 1279 | 20.8 |
| *Rarely* | 0 | 3.1 | 0 | 0.1 | 0 | 0.9 | 0 | 3.2 |
| *Sometimes* | 227 | 3.7 | 9 | 0.1 | 74 | 1.2 | 230 | 3.7 |
| *Often* | 67 | 1.1 | 3 | 0 | 17 | 0.3 | 68 | 1.1 |
| *Always* | 33 | 0.5 | 1 | 0 | 15 | 0.2 | 34 | 0.6 |
| **Reused disposable PPE** | | | | | | | | |
| *No* | 1239 | 20.1 | 35 | 0.6 | 325 | 5.3 | 1260 | 20.5 |
| *Yes* | 537 | 8.7 | 14 | 0.2 | 134 | 2.2 | 546 | 8.9 |
| **Used improvised PPE** | | | | | | | | |
| *No* | 1518 | 24.7 | 40 | 0.7 | 401 | 6.5 | 1540 | 25 |
| *Yes* | 258 | 4.2 | 9 | 0.1 | 58 | 0.9 | 266 | 4.3 |

**eTable 5. Outcomes and composite summary**% out of all participants (n = 6152)

*Abbreviations*: SD = Standard Deviation, BAME = Black, Asian and Minority Ethnic, AGP = Aerosol-Generating Procedures, PPE = Personal Protective Equipment.

|  | **Multivariable adjusted OR** | **95%CI** | ***p*-value** |
| --- | --- | --- | --- |
| **Age** | | | |
| *Age (per year)** | 0.98 | 0.98 - 0.99 | <0.001 |
| **Sex** | | | |
| *Female* | Ref | Ref | Ref |
| *Male* | 0.92 | 0.79 - 1.06 | 0.255 |
| **Ethnicity** | | | |
| *White* | Ref | Ref | Ref |
| *Another ethnic group* | 0.96 | 0.67 - 1.36 | 0.828 |
| *Asian or Asian British* | 0.96 | 0.80 - 1.14 | 0.643 |
| *Black, African, Black British or Caribbean* | 1.03 | 0.79 - 1.33 | 0.845 |
| *Mixed or multiple ethnic groups* | 0.99 | 0.68 - 1.40 | 0.937 |
| *Prefer not to say* | 0.91 | 0.36 - 2.06 | 0.827 |
| **Household - Persons** | | | |
| *Lives alone* | Ref | Ref | Ref |
| *Lives with 1 or more persons; no children* | 0.88 | 0.72 - 1.07 | 0.182 |
| *Lives with 1 or more persons; has children* | 1.00 | 0.83 - 1.21 | 0.961 |
| **Comorbidities** | | | |
| *Hypertension* | 1.13 | 0.91 - 1.40 | 0.260 |
| *Diabetes* | 1.00 | 0.71 - 1.40 | 0.995 |
| *Cancer** | 1.66 | 1.01 - 2.67 | 0.041 |
| *Heart disease* | 1.18 | 0.69 - 1.97 | 0.529 |
| *Immunosuppression* | 0.83 | 0.52 - 1.29 | 0.420 |
| *Respiratory disease** | 1.26 | 1.04 - 1.52 | 0.015 |
| *Renal disease* | 1.08 | 0.49 - 2.23 | 0.840 |
| *Liver disease* | 1.17 | 0.51 - 2.52 | 0.691 |
| *Neurological disease* | 0.88 | 0.49 - 1.52 | 0.659 |
| *Obesity** | 1.31 | 1.10 - 1.56 | 0.003 |
| *Prefer not to say* | 0.94 | 0.58 - 1.48 | 0.780 |
| **Smoking status** | | | |
| *Never smoked* | Ref | Ref | Ref |
| *Current or Ex-smoker within 1 year** | 0.80 | 0.64 - 0.98 | 0.037 |
| *Ex-smoker > 1 year* | 1.10 | 0.94 - 1.27 | 0.231 |
| *Prefer not to say* | 1.14 | 0.68 - 1.87 | 0.604 |
| **Country** | | | |
| *England* | Ref | Ref | Ref |
| *Northern Ireland** | 0.44 | 0.24 - 0.75 | 0.004 |
| *Scotland* | 0.95 | 0.73 - 1.23 | 0.705 |
| *Wales* | 1.17 | 0.84 - 1.62 | 0.345 |
| **Main Healthcare Facility** | | | |
| *Hospital* | Ref | Ref | Ref |
| *Community healthcare facility* | 0.99 | 0.84 - 1.17 | 0.944 |
| *Other* | 0.82 | 0.61 - 1.08 | 0.168 |
| *Social care facility* | 1.17 | 0.80 - 1.68 | 0.402 |
| **Role Group** | | | |
| *Nurses, midwives and associated staff* | Ref | Ref | Ref |
| *Allied health professionals** | 0.81 | 0.69 - 0.96 | 0.016 |
| *Dentists and dental staff** | 0.53 | 0.33 - 0.82 | 0.006 |
| *Doctors** | 1.21 | 1.04 - 1.41 | 0.013 |
| *Other* | 0.99 | 0.79 - 1.24 | 0.924 |
| **Used public transport to travel to work*** | 1.38 | 1.20 - 1.59 | <0.001 |
| **Regular clinical contact with suspected or confirmed COVID-19 patients*** | 1.33 | 1.15 - 1.54 | <0.001 |
| **Regular exposure to AGP(s) performed in suspected or confirmed COVID-19 patients*** | 0.81 | 0.71 - 0.94 | 0.004 |
| **Sufficient training in PPE usage*** | 0.86 | 0.75 - 0.98 | 0.024 |
| **Lacked access to PPE items for clinical contact with suspected or confirmed COVID-19 patients*** | 1.28 | 1.09 - 1.51 | 0.002 |
| **Clinical contact without adequate PPE** | | | |
| *Never* | Ref | Ref | Ref |
| *Rarely* | 1.06 | 0.87 - 1.29 | 0.557 |
| *Sometimes** | 1.70 | 1.37 - 2.10 | <0.001 |
| *Often** | 1.84 | 1.28 - 2.63 | 0.001 |
| *Always** | 2.93 | 1.71 - 5.05 | <0.001 |
| **Reused disposable PPE** | 0.98 | 0.86 - 1.13 | 0.814 |
| **Used improvised PPE*** | 0.81 | 0.68 - 0.97 | 0.020 |

**eTable 6. Multivariable logistic regression model featuring constituent BAME groups**

Multivariate odds ratio (OR), 95% confidence intervals (95%CI)

* *p*-value <0.05

*Abbreviations*: Ref = Reference value, BAME = Black, Asian and Minority Ethnic, AGP = Aerosol-Generating Procedures, PPE = Personal Protective Equipment

|  | **Multivariable adjusted OR** | **95%CI** | ***p*-value** |
| --- | --- | --- | --- |
| **Age** | | | |
| *Age (per year)** | 0.99 | 0.98 - 0.99 | <0.001 |
| **Sex** | | | |
| *Female* | Ref | Ref | Ref |
| *Male* | 0.92 | 0.79 - 1.06 | 0.242 |
| **Ethnicity** | | | |
| *White* | Ref | Ref | Ref |
| *BAME* | 0.98 | 0.85 - 1.12 | 0.756 |
| *Prefer not to say* | 0.90 | 0.36 - 2.03 | 0.804 |
| **Household – Persons** | | | |
| *Lives alone* | Ref | Ref | Ref |
| *Lives with 1 or more persons; no children* | 0.86 | 0.71 - 1.05 | 0.130 |
| *Lives with 1 or more persons; has children* | 0.98 | 0.81 - 1.18 | 0.820 |
| **Smoking status** | | | |
| *Never smoked* | Ref | Ref | Ref |
| *Current or Ex-smoker within 1 year** | 0.79 | 0.64 - 0.98 | 0.034 |
| *Ex-smoker > 1 year* | 1.10 | 0.95 - 1.28 | 0.208 |
| *Prefer not to say* | 1.15 | 0.69 - 1.88 | 0.578 |
| **Country** | | | |
| *England* | Ref | Ref | Ref |
| *Northern Ireland** | 0.44 | 0.24 - 0.75 | 0.004 |
| *Scotland* | 0.96 | 0.74 - 1.24 | 0.783 |
| *Wales* | 1.17 | 0.83 - 1.62 | 0.356 |
| **Main Healthcare Facility** | | | |
| *Hospital* | Ref | Ref | Ref |
| *Community healthcare facility* | 0.99 | 0.84 - 1.17 | 0.913 |
| *Other* | 0.80 | 0.60 - 1.06 | 0.132 |
| *Social care facility* | 1.15 | 0.79 - 1.65 | 0.467 |
| **Role Group** | | | |
| *Nurses, midwives and associated staff* | Ref | Ref | Ref |
| *Allied health professionals** | 0.8 | 0.68 - 0.94 | 0.008 |
| *Dentists and dental staff** | 0.51 | 0.32 - 0.79 | 0.004 |
| *Doctors** | 1.18 | 1.02 - 1.37 | 0.028 |
| *Other* | 1.00 | 0.79 - 1.25 | 0.974 |
| **Used public transport to travel to work*** | 1.38 | 1.19 - 1.58 | <0.001 |
| **Regular clinical contact with suspected or confirmed COVID-19 patients*** | 1.32 | 1.14 - 1.52 | <0.001 |
| **Regular exposure to AGP(s) performed in suspected or confirmed COVID-19 patients*** | 0.82 | 0.71 - 0.94 | 0.004 |
| **Sufficient training in PPE usage*** | 0.85 | 0.74 - 0.97 | 0.019 |
| **Lacked access to PPE items for clinical contact with suspected or confirmed COVID-19 patients*** | 1.28 | 1.09 - 1.50 | 0.002 |
| **Clinical contact without adequate PPE** | | | |
| *Never* | Ref | Ref | Ref |
| *Rarely* | 1.07 | 0.88 - 1.30 | 0.495 |
| *Sometimes** | 1.71 | 1.38 - 2.11 | <0.001 |
| *Often** | 1.85 | 1.29 - 2.65 | 0.001 |
| *Always** | 2.87 | 1.69 - 4.94 | <0.001 |
| **Reused disposable PPE** | 0.99 | 0.86 - 1.13 | 0.859 |
| **Used improvised PPE*** | 0.82 | 0.69 - 0.98 | 0.030 |

**eTable 7. Multivariable logistic regression model with comorbidities removed to assess for collider bias with respect to smoking status**

Multivariate odds ratio (OR), 95% confidence intervals (95%CI)

* *p*-value <0.05

*Abbreviations*: Ref = Reference value, BAME = Black, Asian and Minority Ethnic, AGP = Aerosol-Generating Procedures, PPE = Personal Protective Equipment

|  | **Multivariable adjusted OR** | **95%CI** | ***p*-value** |
| --- | --- | --- | --- |
| **Age** | | | |
| *Age (per year)** | 0.99 | 0.98 - 0.99 | <0.001 |
| **Sex** | | | |
| *Female* | Ref | Ref | Ref |
| *Male* | 0.96 | 0.81 - 1.15 | 0.687 |
| **Ethnicity** | | | |
| *White* | Ref | Ref | Ref |
| *BAME* | 0.97 | 0.82 - 1.16 | 0.770 |
| *Prefer not to say* | 0.99 | 0.32 - 2.72 | 0.980 |
| **Household – Persons** | | | |
| *Lives alone* | Ref | Ref | Ref |
| *Lives with 1 or more persons; no children* | 0.92 | 0.73 - 1.17 | 0.504 |
| *Lives with 1 or more persons; has children* | 1.02 | 0.81 - 1.28 | 0.899 |
| **Comorbidities** | | | |
| *Hypertension* | 1.23 | 0.93 - 1.62 | 0.144 |
| *Diabetes* | 0.92 | 0.59 - 1.41 | 0.707 |
| *Cancer* | 1.77 | 0.86 - 3.59 | 0.114 |
| *Heart disease* | 1.04 | 0.46 - 2.25 | 0.916 |
| *Immunosuppression* | 1.00 | 0.52 - 1.85 | 0.991 |
| *Respiratory disease* | 1.18 | 0.92 - 1.50 | 0.181 |
| *Renal disease* | 1.30 | 0.48 - 3.26 | 0.583 |
| *Liver disease* | 1.42 | 0.51 - 3.69 | 0.480 |
| *Neurological disease* | 0.85 | 0.40 - 1.69 | 0.649 |
| *Obesity** | 1.30 | 1.04 - 1.62 | 0.018 |
| *Prefer not to say* | 0.59 | 0.29 - 1.09 | 0.107 |
| **Smoking status** | | | |
| *Never smoked* | Ref | Ref | Ref |
| *Current or Ex-smoker within 1 year* | 0.83 | 0.64 - 1.07 | 0.161 |
| *Ex-smoker > 1 year* | 1.08 | 0.90 - 1.30 | 0.412 |
| *Prefer not to say* | 1.18 | 0.62 - 2.18 | 0.594 |
| **Country** | | | |
| *England* | Ref | Ref | Ref |
| *Northern Ireland** | 0.42 | 0.21 - 0.76 | 0.007 |
| *Scotland* | 0.88 | 0.63 - 1.21 | 0.431 |
| *Wales* | 1.42 | 0.93 - 2.14 | 0.096 |
| **Main Healthcare Facility** | | | |
| *Hospital* | Ref | Ref | Ref |
| *Community healthcare facility* | 1.20 | 0.86 - 1.69 | 0.288 |
| *Other* | 1.15 | 0.68 - 1.93 | 0.588 |
| *Social care facility* | 0.96 | 0.50 - 1.81 | 0.909 |
| **Role Group** | | | |
| *Nurses, midwives and associated staff* | Ref | Ref | Ref |
| *Allied health professionals* | 0.86 | 0.69 - 1.06 | 0.161 |
| *Dentists and dental staff* | 0.71 | 0.25 - 1.90 | 0.511 |
| *Doctors* | 1.19 | 0.99 - 1.44 | 0.070 |
| *Other* | 0.94 | 0.65 - 1.36 | 0.758 |
| **Used public transport to travel to work*** | 1.28 | 1.08 - 1.52 | 0.005 |
| **Clinical areas where healthcare workers were regularly in clinical contact with suspected or confirmed COVID-19 patients** | | | |
| *covid-19 zone* | 1.09 | 0.93 - 1.27 | 0.274 |
| *Care/nursing home* | 1.16 | 0.77 - 1.72 | 0.476 |
| *Inpatient clinic** | 1.41 | 1.01 - 1.97 | 0.043 |
| *Outpatient clinic* | 1.04 | 0.80 - 1.36 | 0.750 |
| *Day surgery unit* | 0.96 | 0.59 - 1.51 | 0.857 |
| *Emergency department* | 0.96 | 0.80 - 1.15 | 0.640 |
| *Endoscopy* | 0.89 | 0.50 - 1.54 | 0.691 |
| *GP practice* | 0.68 | 0.45 - 1.02 | 0.063 |
| *Home visits** | 0.68 | 0.47 - 0.98 | 0.040 |
| *Intensive care/High dependency unit** | 0.78 | 0.65 - 0.94 | 0.007 |
| *Inpatient ward (non-COVID)* | 1.08 | 0.91 - 1.29 | 0.370 |
| *Maternity* | 0.79 | 0.59 - 1.05 | 0.114 |
| *Operating theatre** | 0.71 | 0.57 - 0.87 | 0.001 |
| *Radiology** | 0.62 | 0.42 - 0.91 | 0.016 |
| *Other** | 0.69 | 0.48 - 0.98 | 0.044 |
| **Regular exposure to AGP(s) performed in suspected or confirmed COVID-19 patients** | 0.95 | 0.79 - 1.14 | 0.571 |
| **Sufficient training in PPE usage*** | 0.83 | 0.70 - 0.99 | 0.043 |
| **Lacked access to PPE items for clinical contact with suspected or confirmed COVID-19 patients*** | 1.30 | 1.09 - 1.57 | 0.004 |
| **Clinical contact without adequate PPE** | | | |
| *Never* | Ref | Ref | Ref |
| *Rarely* | 1.02 | 0.82 - 1.26 | 0.887 |
| *Sometimes** | 1.55 | 1.24 - 1.95 | <0.001 |
| *Often** | 1.53 | 1.04 - 2.24 | 0.029 |
| *Always** | 2.53 | 1.42 - 4.56 | 0.002 |
| **Reused disposable PPE** | 0.97 | 0.83 - 1.14 | 0.731 |
| **Used improvised PPE** | 0.83 | 0.67 - 1.02 | 0.077 |

**eTable 8. Multivariable logistic regression model including clinical areas in which healthcare workers had regular clinical contact with suspected or confirmed COVID-19 patients**

Multivariate odds ratio (OR), 95% confidence intervals (95%CI)

* *p*-value <0.05

*Abbreviations*: Ref = Reference value, BAME = Black, Asian and Minority Ethnic, GP = General practitioner, AGP = Aerosol-Generating Procedures, PPE = Personal Protective Equipment

|  | **Multivariable adjusted OR** | **95%CI** | ***p*-value** |
| --- | --- | --- | --- |
| **Age** | | | |
| *Age** | 0.98 | 0.97 - 0.99 | <0.001 |
| **Sex** | | | |
| *Female* | Ref | Ref | Ref |
| *Male* | 0.95 | 0.76 - 1.20 | 0.681 |
| **Ethnicity** | | | |
| *White* | Ref | Ref | Ref |
| *BAME* | 0.99 | 0.78 - 1.25 | 0.934 |
| *Prefer not to say* | 1.22 | 0.33 - 4.09 | 0.751 |
| **Household - Persons** | | | |
| *Lives alone* | Ref | Ref | Ref |
| *Lives with 1 or more persons; no children* | 0.96 | 0.71 - 1.32 | 0.812 |
| *Lives with 1 or more persons; has children* | 1.23 | 0.91 - 1.67 | 0.189 |
| **Comorbidities** | | | |
| *Hypertension* | 1.30 | 0.90 - 1.88 | 0.159 |
| *Diabetes* | 0.93 | 0.50 - 1.64 | 0.796 |
| *Cancer* | 2.03 | 0.71 - 5.72 | 0.177 |
| *Heart disease* | 0.67 | 0.23 - 1.68 | 0.425 |
| *Immunosuppression* | 1.11 | 0.42 - 2.68 | 0.825 |
| *Respiratory disease* | 0.96 | 0.68 - 1.34 | 0.803 |
| *Renal disease* | 2.39 | 0.87 - 6.31 | 0.080 |
| *Liver disease* | 2.14 | 0.60 - 7.06 | 0.215 |
| *Neurological disease* | 1.16 | 0.38 - 3.14 | 0.779 |
| *Obesity* | 1.14 | 0.85 - 1.53 | 0.383 |
| *Prefer not to say* | 0.59 | 0.19 - 1.51 | 0.310 |
| **Smoking status** | | | |
| *Never smoked* | Ref | Ref | Ref |
| *Current or Ex-smoker within 1 year* | 1.03 | 0.74 - 1.42 | 0.841 |
| *Ex-smoker > 1 year* | 1.03 | 0.79 - 1.32 | 0.839 |
| *Prefer not to say* | 0.87 | 0.32 - 2.10 | 0.760 |
| **Country** | | | |
| *England* | Ref | Ref | Ref |
| *Northern Ireland** | 0.41 | 0.16 - 0.90 | 0.039 |
| *Scotland* | 0.77 | 0.48 - 1.22 | 0.285 |
| *Wales* | 1.29 | 0.76 - 2.14 | 0.331 |
| **Main Healthcare Facility** | | | |
| *Hospital* | Ref | Ref | Ref |
| *Community healthcare facility* | 1.72 | 0.94 - 3.14 | 0.078 |
| *Other* | 1.50 | 0.76 - 2.92 | 0.235 |
| *Social care facility* | 1.23 | 0.21 - 6.34 | 0.804 |
| **Role Group** | | | |
| *Nurses, midwives and associated staff* | Ref | Ref | Ref |
| *Allied health professionals** | 0.74 | 0.56 - 0.98 | 0.039 |
| *Dentists and dental staff* | 0.39 | 0.14 - 1.05 | 0.062 |
| *Doctors* | 1.16 | 0.90 - 1.50 | 0.258 |
| *Other** | 1.83 | 1.03 - 3.21 | 0.037 |
| **Used public transport to travel to work*** | 1.37 | 1.09 - 1.72 | 0.007 |
| **Regular clinical contact with suspected or confirmed COVID-19 patients** | 1.01 | 0.68 - 1.53 | 0.943 |
| **AGPs to which healthcare workers were regularly exposed when performed in suspected or confirmed COVID-19 patients** | | | |
| *Bronchoscopy and ENT procedures* | 0.95 | 0.75 - 1.21 | 0.705 |
| *Dental procedures* | 0.87 | 0.45 - 1.61 | 0.676 |
| *Cardiopulmonary resuscitation* | 0.81 | 0.62 - 1.06 | 0.127 |
| *High-flow nasal oxygen* | 1.17 | 0.93 - 1.48 | 0.170 |
| *High frequency oscillatory ventilation* | 1.15 | 0.75 - 1.72 | 0.516 |
| *Induction of sputum* | 1.19 | 0.91 - 1.55 | 0.192 |
| *Intubation/Extubation* | 0.80 | 0.63 - 1.00 | 0.054 |
| *Nebulisers* | 0.96 | 0.75 - 1.22 | 0.722 |
| *Non-invasive ventilation* | 1.02 | 0.81 -1.28 | 0.884 |
| *Surgery and post-mortem* | 0.94 | 0.72 - 1.22 | 0.637 |
| *Tracheostomy* | 0.80 | 0.63 - 1.00 | 0.051 |
| *Endoscopy* | 0.88 | 0.60 - 1.27 | 0.510 |
| *Other* | 0.83 | 0.55 - 1.24 | 0.371 |
| **Sufficient training in PPE usage** | 0.82 | 0.63 - 1.07 | 0.135 |
| **Lacked access to PPE items for clinical contact with suspected or confirmed COVID-19 patients*** | 1.41 | 1.10 - 1.81 | 0.007 |
| **Clinical contact without adequate PPE** | | | |
| *Never* | Ref | Ref | Ref |
| *Rarely* | 1.17 | 0.87 - 1.56 | 0.286 |
| *Sometimes** | 1.53 | 1.10 - 2.13 | 0.011 |
| *Often* | 1.25 | 0.74 - 2.08 | 0.400 |
| *Always** | 3.82 | 1.68 - 9.11 | 0.002 |
| **Reused disposable PPE** | 0.91 | 0.74 - 1.12 | 0.370 |
| **Used improvised PPE** | 0.82 | 0.62 - 1.08 | 0.169 |

**eTable 9. Multivariable logistic regression model including AGPs to which healthcare workers were regularly exposed to when performed in suspected or confirmed COVID-19 patients**

Multivariate odds ratio (OR), 95% confidence intervals (95%CI)

* *p*-value <0.05

*Abbreviations*: Ref = Reference value, BAME = Black, Asian and Minority Ethnic, AGP = Aerosol-Generating Procedures, ENT = Ear, Nose and Throat, PPE = Personal Protective Equipment

|  | **Multivariable adjusted OR** | **95%CI** | ***p*-value** |
| --- | --- | --- | --- |
| **Age** | | | |
| *Age* | 1.00 | 0.99 - 1.01 | 0.907 |
| **Sex** |  |  |  |
| *Female* | Ref | Ref | Ref |
| *Male* | 1.24 | 0.97 - 1.57 | 0.077 |
| **Ethnicity** |  |  |  |
| *White* | Ref | Ref | Ref |
| *BAME* | 0.99 | 0.77 - 1.25 | 0.914 |
| *Prefer not to say* | 0.41 | 0.02 - 2.04 | 0.391 |
| **Household - Persons** |  |  |  |
| *Lives alone* | Ref | Ref | Ref |
| *Lives with 1 or more persons; no children* | 1.00 | 0.71 - 1.41 | 0.983 |
| *Lives with 1 or more persons; has children* | 1.03 | 0.75 - 1.45 | 0.844 |
| **Comorbidities** |  |  |  |
| *Hypertension* | 0.89 | 0.59 - 1.29 | 0.548 |
| *Diabetes* | 1.14 | 0.64 - 1.93 | 0.636 |
| *Cancer* | 0.96 | 0.33 - 2.23 | 0.932 |
| *Heart disease* | 0.77 | 0.23 - 1.94 | 0.630 |
| *Immunosuppression** | 0.23 | 0.04 - 0.74 | 0.042 |
| *Respiratory disease* | 1.17 | 0.84 - 1.60 | 0.324 |
| *Renal disease* | 1.59 | 0.37 - 4.60 | 0.455 |
| *Liver disease* | 0.82 | 0.13 - 2.92 | 0.792 |
| *Neurological disease* | 0.66 | 0.16 - 1.83 | 0.492 |
| *Obesity* | 0.94 | 0.67 - 1.29 | 0.727 |
| *Prefer not to say* | 1.10 | 0.48 - 2.21 | 0.811 |
| **Smoking status** |  |  |  |
| *Never smoked* | Ref | Ref | Ref |
| *Current or Ex-smoker within 1 year** | 0.59 | 0.38 - 0.88 | 0.014 |
| *Ex-smoker > 1 year* | 1.10 | 0.85 - 1.41 | 0.481 |
| *Prefer not to say* | 1.61 | 0.73 - 3.18 | 0.198 |
| **Country** |  |  |  |
| *England* | Ref | Ref | Ref |
| *Northern Ireland* | 0.74 | 0.28 - 1.58 | 0.478 |
| *Scotland* | 0.74 | 0.43 - 1.18 | 0.228 |
| *Wales* | 1.29 | 0.72 - 2.17 | 0.358 |
| **Main healthcare facility** |  |  |  |
| *Hospital* | Ref | Ref | Ref |
| *Community healthcare facility** | 0.66 | 0.47 - 0.90 | 0.010 |
| *Other* | 0.58 | 0.29 - 1.04 | 0.087 |
| *Social care facility* | 1.27 | 0.68 - 2.23 | 0.431 |
| **Role group** |  |  |  |
| *Nurses, midwives and associated staff* | Ref | Ref | Ref |
| *Allied health professionals** | 0.63 | 0.45 - 0.85 | 0.004 |
| *Dentists and dental staff* | 0.27 | 0.04 - 0.94 | 0.083 |
| *Doctors* | 1.21 | 0.95 - 1.55 | 0.126 |
| *Other* | 1.19 | 0.79 - 1.77 | 0.393 |
| **Used public transport to travel to work** | 0.98 | 0.76 - 1.25 | 0.870 |
| **Regular clinical contact with suspected or confirmed COVID-19 patients*** | 2.18 | 1.66 - 2.89 | <0.001 |
| **Regular exposure to AGP(s) performed in suspected or confirmed COVID-19 patients*** | 0.65 | 0.52 - 0.81 | <0.001 |
| **Sufficient training in PPE usage** | 1.14 | 0.90 - 1.46 | 0.274 |
| **Lacked access to PPE items for clinical contact with suspected or confirmed COVID-19 patients*** | 1.67 | 1.28 - 2.16 | <0.001 |
| **Clinical contact without adequate PPE** |  |  |  |
| *Never* | Ref | Ref | Ref |
| *Rarely* | 1.13 | 0.81 - 1.56 | 0.445 |
| *Sometimes** | 1.79 | 1.29 - 2.45 | <0.001 |
| *Often* | 1.39 | 0.77 - 2.38 | 0.248 |
| *Always** | 3.71 | 1.89 - 6.94 | <0.001 |
| **Reused disposable PPE** | 0.86 | 0.68 - 1.08 | 0.203 |
| **Used improvised PPE*** | 0.63 | 0.45 - 0.85 | 0.003 |

**eTable 10. Multivariable logistic regression model with the outcome being participants stating that they had laboratory-confirmed SARS-CoV-2 infection (via reverse transcription polymerase chain reaction or antibody testing)**

Multivariate odds ratio (OR), 95% confidence intervals (95%CI)

* *p*-value <0.05

*Abbreviations*: Ref = Reference value, BAME = Black, Asian and Minority Ethnic, AGP = Aerosol-Generating Procedures, ENT = Ear, Nose and Throat, PPE = Personal Protective Equipment
