# Supplementary material for: healthcareCOVID: a national cross-sectional observational study identifying risk factors for developing suspected or confirmed COVID-19 in UK healthcare workers
Source: PeerJ. 2021 Feb 4;9:e10891. doi: 10.7717/peerj.10891 (PMC7868068; doi:10.7717/peerj.10891)
Supplement: Supplemental Information 4 [file peerj-09-10891-s004.pdf]

Guy's and St Thomas' NHS Foundation Trust

healthcareCOVID

Chief Investigator:
Dr Kariem El-Boghdadly
Department of Anaesthesia, Guy's and St Thomas' NHS Foundation Trust, London, UK

Lead Site:
Guy's and St Thomas' (GSTT) NHS Foundation Trust

GSTT Service Evaluation ID: *10834*

Protocol Version Number and Date:
Version **1.0**
(04/05/2020)

Study Summary:

**Full Scientific Title**	HealthcareCOVID
**Health Conditions(s) or problems(s) being studied**	Risk factors and incidence of Covid-19 infection amongst healthcare workers.
**Study type**	Service evaluation, via snapshot survey
**Target sample size**	>1000

Study Timelines:

**Study Duration**	3 months
**Expected start date**	26/04/2020
**End of study definition**	Data collection for 14 days
**Key study milestones:**	• Study set up: April 2020<br>• Study start: 04/05/2020<br>• Study completion: 18/05/2020<br>• Analysis and Write Up: June/July 2020

Key Study Contacts:

**Chief Investigator**	**Dr Kariem El-Boghdadly**<br>*Address:* Department of Anaesthesia, Guy's and St Thomas' NHS Foundation Trust, London, UK<br>*Email:* kariem.elboghdadly@gstt.nhs.net
**Co – Investigator**	**Dr Imran Ahmed**<br>*Address:* Department of Anaesthesia, Guy's and St Thomas' NHS Foundation Trust, London, UK<br>*Email:* imran.ahmad@gstt.nhs.uk<br><br>**Dr Calvin Moorley**<br>*Address:* School of Health and Social Care/Adult Nursing & Midwifery Studies, London South Bank University, 103 Borough Road, London SE1 0AA<br>*Email:* moorleyc@LSBU.ac.uk
**Trainee Investigators**	**Dr Justin Kua – Trainee Lead Investigator**<br>*Address*: UCL Surgical Outcomes Research Centre and Centre for Perioperative Medicine; Charles Bell House, 43-47 Foley Street, London W1T 7SG<br>*E-mail:* justin.kua@nhs.net<br><br>**Dr Danny Wong**<br>*Address:* Department of Anaesthesia, Guy's and St Thomas' NHS Foundation Trust, London, UK<br>*Email:* dannywong@doctors.org.uk<br><br>**Dr Reshma Patel**<br>*Address*: UCL Surgical Outcomes Research Centre and Centre for Perioperative Medicine; Charles Bell House, 43-47 Foley Street, London W1T 7SG<br>*E-mail*: reshma.patel@ucl.ac.uk

INTRODUCTION

Coronavirus infection 2019 (COVID-19), caused by severe acute respiratory syndrome coronavirus 2 (SARS-CoV-2), has resulted in a global health crisis which has challenged healthcare systems around the world. According to the World Health Organization (WHO), there are over 2.8 million confirmed cases with over 193,000 deaths worldwide as of 26th April 2020.[1] Healthcare workers (HCWs) around the world are central to combating COVID-19, especially those working on the frontlines to diagnose and treat COVID-19 patients. Unfortunately, despite efforts to protect HCWs with protocols and use of personal protective equipment (PPE), this cohort still remains susceptible to the full spectrum of severity of COVID-19 infection. In China, over 3,300 HCWs were revealed to have been infected with COVID-19 as of early March.[2,3] In Italy, 4,824 HCWs have been infected, accounting for 9% of all COVID-19 cases. In the UK, an analysis of news and social media reports revealed a total of 119 deaths of HCWs infected with COVID-19 working within the National Health Service (NHS) as of 22nd April 2020.[4]

The incidence of COVID-19 in HCWs is thought to be higher than the general population due to exposure to higher viral loads from increased contact with infected individuals.[5,6] Due to the nature of SARS-CoV-2 transmission, i.e. via respiratory droplets, it is thought that HCWs exposed to aerosol generating procedures (AGPs) are at higher risk of developing COVID-19 infection.[6] In an effort to study the link between HCWs exposed to AGPs, PPE use and consequent COVID-19 infection, "intubateCOVID" was developed and has been well-received in several countries.[7] This unique registry is an ongoing international effort to collect data on all intubation episodes and symptom development in airway mangers and assistants. A similar registry has been created for maternity services, "obsCOVID", which explores encounters with suspected of confirmed COVID-19 parturients and consequent COVID-19 infection in maternity-/labour ward-based HCWs. However, both registries are limited to HCWs working within each respective environment and unfortunately do not have the breadth to capture the greater population of HCWs who have differing levels of exposure based on other working environments and use of PPE. Indeed, HCWs working in primary care, such as general practitioners (GPs) and community nurses, are potentially more exposed to milder forms of the disease and still bear the risk of contracting mild to severe COVID-19.

Efforts to increase screening of HCWs for COVID-19 infection have been ongoing with attempts by the UK government to ramp up testing since early April. There have been 2 published articles of COVID-19 screening of HCWs within the NHS. Keeley et al.[8] tested 1,533 staff members with influenza-like illness in Sheffield Teaching Hospitals NHS Foundation Trust and found 282 positive tests, an overall prevalence of 18%. A limitation of data collection was the lack of symptoms recorded and the roles of staff members which precluded further stratification. A similar testing strategy was used in Newcastle upon Tyne Hospitals NHS Foundation Trust, identifying 240 positive tests in 1,654 staff members tested (14% prevalence rate).[9] 1,029 staff members had data on staff roles and were stratified into directly patient facing, non-patient facing but potentially at higher risk of nosocomial exposure, and non-clinical. There was no significant difference between groups in the rates of COVID-19 infection. However, like the previous study, there was limited data on all staff roles and no data on symptoms. In addition, both studies were limited to the hospital environment, thus failing to capture HCWs working within the community.

Recent attention has been shed on the prominence of black, Asian and minority ethnic (BAME) groups with regard to severity of COVID-19 infection.[4,10,11] A recent report from the Intensive Care National Audit & Research Centre (ICNARC) revealed a higher proportion of critically ill BAME individuals versus other ethnicities with relation to confirmed COVID-19 when compared to non-COVID-19 viral pneumonia.[12] Specifically looking at HCWs, an analysis of news and social media reports of deaths related to COVID-19 in HCWs by Cook et al. showed that 63% of cases were of

BAME origin.[4] HCW deaths were not limited to those working in hospitals but were also seen in community HCWs. The reasons for this preponderance of BAME groups and COVID-19 infection in HCWs as well as the general population are likely to be multifactorial.[11] Nonetheless, further investigation is required to examine the relationship between HCW demographics and working environment and COVID-19 infection.

HealthcareCOVID is a national, cohort, snapshot service evaluation project aimed at investigating the risk factors associated with exposure and consequent infection with COVID-19 in the HCW population of UK. The study cohort will encompass HCWs involved in primary and secondary care, including doctors, nurses and allied health professionals (AHPs). An online questionnaire will facilitate data collection directly from each HCW. This questionnaire, which covers the period from 1st February 2020 to currently, will comprise basic demographic data, environmental exposure to COVID-19 and PPE use. The results of this service evaluation will inform us of the prevalence and impact of COVID-19 on HCWs, establish a baseline covering the period from 1st Feb 2019 up until current day (signified by the end of the snapshot period), and provide better information and comparability for colleagues, planners, and strategists regarding this topic.

OBJECTIVES AND PURPOSE

Aims

1. To establish a baseline prevalence of suspected (i.e. symptomatic self-isolation) and confirmed (i.e. through SARS-CoV-2 testing) COVID-19 in HCWs from 1st Feb 2019 to present day.

2. To evaluate the risk factors for HCWs in the development of COVID-19 infection.

3. To provide information to relevant agencies involved in the planning of public health services with regards to protecting HCWs throughout the COVID-19 pandemic

Service Evaluation Questions

1. ***SEQ1*** - What is the prevalence of suspected and confirmed COVID-19 infection amongst healthcare workers?

2. ***SEQ2*** – What risk factors are associated with COVID-19 infection amongst healthcare workers, stratified by demographics (including ethnicity), working environment and PPE use?

3. ***SEQ3*** – Is current PPE practice adequate in limiting the rates of COVID-19 infection amongst healthcare workers with regards to exposure to suspected/confirmed COVID-19 cases?

STUDY DESIGN

HealthcareCOVID is a national, cohort, snapshot service evaluation project that will utilise an online questionnaire to investigate the risk factors associated with exposure and consequent suspected or confirmed infection with COVID-19 in HCWs working within the UK. Any HCW above the age of 18 years and working in a UK-based healthcare facility (e.g. hospital, clinic, GP practice, care/nursing home) will be eligible to participate. The service evaluation will utilise a snapshot-styled method of

data collection, occurring over a maximum period of 2 weeks. It should be noted that this service evaluation project is not considered 'research' as per the Health Research Authority's decision tool (see ***Appendix 2***).

Data collection

Participants who wish to be involved in healthcareCOVID will need to follow a link to a secure, encrypted data entry form at https://intubatecovid.knack.com/healthcarecovid. Completed questionnaires will be stored on a web-based database developed by the study team, with the chief investigator based at Guy's and St Thomas' NHS Foundation Trust (GSTT). The database will be located on a secure cloud-based server in Frankfurt and maintained by Knack (Philadelphia, USA). Knack, the online software used for data capture from participants, is a secure web application for building and managing online databases. Knack is compliant with the General Data Protection Regulation (https://www.knack.com/gdpr).

Participants will be directed to the survey via a uniform resource locator (URL) link. Responses will be entered via a secure, encrypted connection onto a secure online portal hosted by Knack. The snapshot survey will comprise:

- A valid e-mail address to reduce the chance of duplicate entries and to allow later communication of results, should the participant so choose
- Details of the participants role within the healthcare system, including job, grade, specialty and healthcare institution
- Basic demographic data, including age, ethnicity and basic health status
- Details on self-isolation periods due to symptoms linked with possible or confirmed COVID-19 infection, hospitalisation, and results of positive COVID-19 testing
- Details of exposure to COVID-19 during day-to-day work, particularly focused on those having direct clinical contact with suspected or confirmed COVID-19 cases and use of AGPs
- PPE use and availability

A summary of planned questions can be found in ***Appendix 3***. Apart from the participant's e-mail address (which will not be linked to entries during data analysis), no other user-identifiable data will be collected. Collected data will be used to evaluate current practice with regard to clinical contact with patients with suspected or confirmed COVID-19 infection and PPE use, and to identify clinician provider health outcomes following such clinical contact (if any).

Inclusion & Exclusion Criteria

Inclusion criteria

- Any HCW – including doctors, nurses, and all AHP working in the UK, in primary or secondary care services since 1st February 2020.
- 18 years of age or above

Exclusion criteria

- HCWs who have not practiced clinical work since 1st February 2020.
- HCWs practicing outside the UK.
- HCWs under the age of 18 years

Enrolment Process

The snapshot questionnaire will be available via a weblink. This link will be made available to HCWs via their respective official colleges, and open access to the link via social media. We plan to involve several HCW organisations such as the Academy of Medical Royal Colleges (AoMRC), the Allied Health Professions Federation (AHPF), and the Royal College of Nursing and Midwifery, as well as

other relevant bodies (including other Royal Colleges within the UK) to aid dissemination. Following commencement of the snapshot period, HCWs will be able to access the survey for immediate data entry up until closure.

Participant Withdrawal
No HCW is obliged to take part and completion of the questionnaire is voluntary. However, if a participant chooses to withdraw after the questionnaire is completed, it will not be possible to remove their data as analysis will have already commenced.

Consent
Participation in the study by filling in and completing the questionnaire is interpreted as consent to take part. A link to a disclaimer (see ***Appendix 4***) is available for viewing by participants prior to completion of the survey. No personal data or sensitive information is linked with responses during data analysis. The study will be openly advertised via social media and e-mails of relevant healthcare worker organisations.

Data Handling and Management
All investigators and study staff will be expected to comply with the requirements of the Data Protection Act 2018 with regard to the collection, storage, processing and disclosure of personal information and will uphold the Act's core principles.

Data from the questionnaire is submitted to the central repository and will only be accessible by the study investigators. The e-mail address supplied by each participant will only be used to prevent duplicate entries and will not be linked to responses during data analysis. Otherwise, no direct user-identifiers will be collected; specifically, there will be no collection of names, dates of birth, addresses, or other identifiers.

Further details on data encryption can be found here:
https://www.knack.com/tour/security#backups.

Reporting and dissemination
Where possible, we plan to present the results of the service evaluation in peer-reviewed journals or other conference presentations to communicate findings to the community and provide updates on potential best practices for personnel risk mitigation. We will also provide summary reports to relevant stakeholder organisations (e.g. AoMRC, Royal Colleges, societies, federations, and groups) and to participants who wish to be informed (as part of their response to the questionnaire).

REFERENCES

1. WHO COVID-19 Dashboard. https://who.sprinklr.com/. Accessed April 18, 2020.

2. The Lancet. COVID-19: protecting health-care workers. *The Lancet*. 2020;395(10228):922. doi:10.1016/S0140-6736(20)30644-9

3. Anelli F, Leoni G, Monaco R, et al. Italian doctors call for protecting healthcare workers and boosting community surveillance during covid-19 outbreak. *BMJ*. 2020;368:m1254. doi:10.1136/bmj.m1254

4. Cook T, Kursumovic E, April 2020 SL. Exclusive: deaths of NHS staff from covid-19 analysed. Health Service Journal. https://www.hsj.co.uk/exclusive-deaths-of-nhs-staff-from-covid-19-analysed/7027471.article. Accessed April 26, 2020.

5. SARS-CoV-2 viral load and the severity of COVID-19. CEBM. https://www.cebm.net/covid-19/sars-cov-2-viral-load-and-the-severity-of-covid-19/. Accessed April 18, 2020.

6. Wilson NM, Norton A, Young FP, Collins DW. Airborne transmission of severe acute respiratory syndrome coronavirus-2 to healthcare workers: a narrative review. *Anaesthesia*. n/a(n/a). doi:10.1111/anae.15093

7. intubateCOVID Team. intubateCOVID. https://intubatecovid.knack.com/registry#project-details/. Accessed April 11, 2020.

8. Keeley AJ, Evans C, Colton H, et al. Roll-out of SARS-CoV-2 testing for healthcare workers at a large NHS Foundation Trust in the United Kingdom, March 2020. *Euro Surveill Bull Eur Sur Mal Transm Eur Commun Dis Bull*. 2020;25(14). doi:10.2807/1560-7917.ES.2020.25.14.2000433

9. Hunter E, Price DA, Murphy E, et al. First experience of COVID-19 screening of health-care workers in England. *The Lancet*. 2020;0(0). doi:10.1016/S0140-6736(20)30970-3

10. Laurencin CT, McClinton A. The COVID-19 Pandemic: a Call to Action to Identify and Address Racial and Ethnic Disparities. *J Racial Ethn Health Disparities*. April 2020. doi:10.1007/s40615-020-00756-0

11. Khunti K, Singh AK, Pareek M, Hanif W. Is ethnicity linked to incidence or outcomes of covid-19? *BMJ*. 2020;369:m1548. doi:10.1136/bmj.m1548

12. House N, Holborn H, Wc L. ICNARC report on COVID-19 in critical care. 2020:26.

LIST OF PROTOCOL APPENDICES

APPENDIX 1 – Protocol amendment / Revision history

APPENDIX 2 – HRA Decision Making Tool Result

APPENDIX 3 – Planned Questions for Service Evaluation → [Awaiting finalisation]

APPENDIX 4 – Disclaimer

APPENDIX 1 – Protocol amendment / Revision history

**Version Stage**	**Versions No.**	**Version Date**	**Protocol updated & finalised by**	**Appendix No. Detail the reason(s) for the protocol update**
Baseline Draft	**1.0**	04/05/2020	Dr Kariem El-Boghdadly<br>Dr Imran Ahmad<br>Dr Justin Kua<br>Dr Reshma Patel<br>Dr Danny Wong	Baseline.

Go straight to content.

Medical Research Council

NHS Health Research Authority

Is my study research?

To print your result with title and IRAS Project ID please enter your details below:

Title of your research:

healthcareCOVID

IRAS Project ID (if available):

N/A

You selected:

- **'No'** - Are the participants in your study randomised to different groups?
- **'No'** - Does your study protocol demand changing treatment/ patient care from accepted standards for any of the patients involved?
- **'No'** - Are your findings going to be generalisable?

Your study would NOT be considered Research by the NHS.

You may still need other approvals.

Researchers requiring further advice (e.g. those not confident with the outcome of this tool) should contact their R&D office or sponsor in the first instance, or the **HRA** to discuss your study. If contacting the HRA for advice, do this by sending an outline of the project (maximum one page), summarising its purpose, methodology, type of participant and planned location as well as a copy of this results page and a summary of the aspects of the decision(s) that you need further advice on to the HRA Queries Line at **Queries@hra.nhs.uk**.

For more information please visit the **Defining Research** table.

Follow this link to start again.

Print This Page

NOTE: If using Internet Explorer please use browser print function.

About this tool **Feedback** **Contact** **Glossary**

APPENDIX 3 – Planned Questions for Service Evaluation

The following is an overview of the data being collected via the online survey:

Entry Validation

- E-mail Address
  - Survey validation check to reduce number of duplicate entries from the same user
  - Will **not** be linked to responses during data analysis
- Option for users to be contacted in future re: findings from survey (optional)

Demographics

- Age
- Sex & Gender identity
- Ethnicity
- Household environment – Occupants and children
- Health Status
  - Health problems, including treated or under control (optional)
  - Tobacco smoking status (optional)

Work Details

- Country
- County/Region
- Main healthcare facility
- Role
- Grade
- Primary specialty – Doctors and Non-Doctors

COVID-19 status (since 1st Feb 2020)

- Self-isolation due to COVID-19 symptoms
  - Duration
  - Repeated self-isolation
- Symptoms during self-isolation
- Hospitalisation due to COVID-19 symptoms
- Positive test for COVID-19

Exposure characteristics (since 1st Feb 2020)

- Use of public transport
- Had regular clinical contact with suspected/confirmed COVID-19 patients?
  - In what areas/environments?
- Had regular exposure to aerosol generating procedures (AGPs) performed in suspected/confirmed COVID-19 patients?
  - What AGPs?

Personal protective equipment (PPE) use (since 1st Feb 2020)

- Lack of access to PPE
- Clinical contact *without* adequate PPE
  - Frequency?
  - Reason(s)
- Reuse of disposable PPE
- Custom-made PPE

Other comments

- Free-text entry

APPENDIX 4 – Disclaimer

Disclaimer

The **healthcareCOVID** project has been registered as a service evaluation (ID 10834) at Guy's and St Thomas' NHS Foundation Trust (London, UK).

By submitting my responses, I confirm that:

1. I understand the purpose of this survey.
2. I have freely chosen to participate.
3. I understand that there will be no reimbursement for my participation.
4. I understand that the principal purpose of this project is to prevent disease transmission and to improve public health services. I understand that communication of findings from this project may involve presentation or publication of collected data and that all data will be de-identified and reported in aggregate.
5. I understand that my data will be stored in an online encrypted database accessed only by the healthcareCOVID team, and in accordance with GDPR principles, and that no personal information will be shared with any third party.
6. I will receive information as requested, only if I have confirmed as such on the survey.
